# Morphology and molecular phylogeny reveal five new species of *Hydnellum* (Bankeraceae, Thelephorales) from China

**DOI:** 10.3389/fmicb.2022.1049007

**Published:** 2022-11-09

**Authors:** Chang-Ge Song, Yi-Fei Sun, Dong-Mei Wu, Neng Gao, Shun Liu, Tai-Min Xu, Bao-Kai Cui

**Affiliations:** ^1^School of Ecology and Nature Conservation, Institute of Microbiology, Beijing Forestry University, Beijing, China; ^2^Xinjiang Production and Construction Group Key Laboratory of Crop Germplasm Enhancement and Gene Resources Utilization, Biotechnology Research Institute, Xinjiang Academy of Agricultural and Reclamation Science, Shihezi, China

**Keywords:** Bankeraceae, ectomycorrhizal fungi, multi-gene phylogeny, stipitate hydnoids, taxonomy

## Abstract

The genus *Hydnellum* is a kind of ectomycorrhizal fungi that can play a role in the material cycle by connecting the plant roots to the soil, and some species of *Hydnellum* are medicinal fungi with vital research value. The species diversity of *Hydnellum* is unclear in China. In this study, five new species of *Hydnellum* are described from China based on morphological characters and phylogenetic analyses inferred from two datasets of ITS + LSU and ITS + LSU + SSU + RPB2 sequences. *H. chocolatum* is characterized by its chocolate basidiomata with the fibrillose, spongy to tomentose pileal surface, and subglobose to globose basidiospores measuring (4.5–)5–6 × 4–5(–5.8) μm. *H. concentricum* is characterized by its zonate pileal surface, thin context, short stipe, presence of both simple septa and clamp connections in generative hyphae of spines, and subglobose to ellipsoidal basidiospores measuring (3.5–)4–5(–5.2) × (3.2–)3.5–5 μm. *H. crassipileatum* is characterized by its thick pileus with the reddish brown to grayish brown pileal surface, and subglobose to ellipsoidal basidiospores measuring 4–6(–6.5) × 4–5.5 μm. *H. melanocarpum* is characterized by its vinaceous brown to black pileus with spongy pileal surface, presence of both simple septa and clamp connections in generative hyphae of spines, and subglobose basidiospores measuring 4.5–5.5(–6) × (3.5–)3.8–5.1 μm. *H. radiatum* is characterized by its radially aligned stripes on pileal surface, grayish brown context, short stipe, and subglobose to ellipsoidal basidiospores measuring (3.5–)4–5 × 3–4.5(–5) μm. Full descriptions, illustrations, and phylogenetic trees to show the placement of the new species are provided.

## Introduction

Stipitate hydnoid fungi of the family Bankeraceae are ectomycorrhizal symbionts of trees in a broad spectrum of forests (Holec and Kučera, 2018), which can provide nutrients and water to roots by exchanging photosynthates from trees and improve the absorption capacity of trees to soil nutrients such as phosphorous (Erland and Taylor, 1999; Parfitt et al., 2007). In some forests, fewer ectomycorrhizal fungi have produced basidiomata (Arnolds, 1991, 2010) and become the emphasis of conservation in Europe (Parfitt et al., 2007). And some species are medicinal fungi, such as *H. concrescens* (Pers.) Banker which has an inhibitory action on syncytium formation, trafficking of glycoprotein and hemagglutinin-neuraminidase (HN) to the cell surface (Lee et al., 2012).

*Hydnellum* P. Karst., typed by *H. suaveolens* (Scop.) P. Karst., is a member of stipitate hydnoids. The genus *Hydnellum* together with *Phellodon* P. Karst. and *Sarcodon* Quél. ex P. Karst was affiliated to Bankeraceae Donk of Thelephorales Corner ex Oberw. The genus *Hydnellum* was established by Karsten (1879). At first, many species of the Bankeraceae including *Hydnellum* were originally classified into the Hydnaceae because of their dentate hymenium (Banker, 1906). Donk established the family Bankeraceae, which consisted of only two genera *Bankera* and *Phellodon* (Donk, 1961). Jülich (1981) revised the classification system of Basidiomycetes and classified *Hydnellum* into the Bankeraceae. The genus *Hydnellum* is characterized by annual basidiomata with a zonate or an azonate pileal surface, spinous and white to orange, gray blue, light brown, or dark brown spines, and centrically or eccentrically stipitate; a monomitic hyphal system with simple septa or clamped generative hyphae, and subglobose to globose and tuberculate basidiospores (Baird and Khan, 1986; Baird et al., 2013). *Hydnellum* is often confused with *Phellodon* and *Sarcodon* because of the similar basidiomata with a hymenium made up of spines (Baird et al., 2013). While the basidiospores in the *Phellodon* species are hyaline, the basidiospores in *Hydnellum* and *Sarcodon* are yellow to brown-tinted (Maas Geesteranus, 1975). Besides, *Sarcodon* differs from *Hydnellum* mainly by its brittle fleshy substance (Banker, 1906) and larger basidiospores (7.4-9 μm; Larsson et al., 2019).

Morphological features, including macroscopic morphological and microscopic morphological characteristics, were commonly used to identify *Hydnellum* species in the past (Banker, 1906; Maas Geesteranus, 1962, 1971; Harrison, 1964; Hrouda, 1999). Banker (1906) conducted a study of hydnaceous fungi of the Czech Republic and Slovakia, and 11 species of *Hydnellum* were newly described. Harrison (1964) conducted a systematic study of the stipitate hydnoids of the Bankeraceae from North America and described 10 species of *Hydnellum*. However, traditional morphology-based generic restrictions are ambiguous (Larsson et al., 2019). Mycologists have used morphological characters and phylogenetic analyses to study the taxonomy of *Hydnellum* in recent years (Ainsworth et al., 2010; Baird et al., 2013; Larsson et al., 2019; Mu et al., 2021). Baird et al. (2013) reevaluated the species of stipitate hydnoids from the southern United States, and 41 distinct taxa were determined including 19 species of *Hydnellum*. Larsson et al. (2019) reassessed the generic limits for *Hydnellum* and *Sarcodon*, and transferred 12 species from *Sarcodon* to *Hydnellum* based on ITS and nLSU sequences, which make the division of the genera clearer. Currently, about 70 species have been described and transferred to the genus according to the records in Index Fungorum (Accessed 7 May 2022)^1^. In recent years, the genus has been studied in China. Mu et al. (2021) described 11 new species from China based on morphological characters and multi-gene phylogenetic analysis.

Species in Bankeraceae are associated with coniferous trees in forest ecosystems and are widely distributed in the northern hemisphere. Stipitate hydnoids were often found in forests on mesic to dry, sandy to loamy soils with, at the most, a thin humus and litter layer (Arnolds, 2010). According to a survey conducted in the Netherlands about 22 species of hydnoid fungi, 12 are associated with deciduous trees older than 40 years, mainly *Quercus robur*, *Quercus rubra*, and *Fagus sylvatica*, and 10 are associated with coniferous trees, almost exclusively with Scots pine (*Pinus sylvestris*) (Arnolds, 2003). However, herb-rich spruce (*Picea abies*) forests on more or less calcareous soils rich in minerals constitute a third important habitat for hydnoid fungi (Arnolds, 2010). These three types of hosts corresponded well in our investigation (Table 2).

Macrofungi have important ecological and economical values. The species diversity, taxonomy, and phylogeny of macrofungi have been extensively investigated in recent years, and many new species have been discovered (Han et al., 2016; Cui et al., 2019; Mu et al., 2019, 2021; Shen et al., 2019; Sun et al., 2020; Cao et al., 2021; Deng et al., 2021, 2022; Liu et al., 2021a,b, 2022a,2022b; Song et al., 2021, 2022; Zhang et al., 2021; Ji et al., 2022; Wang et al., 2022). During our investigations on macrofungi from China, 90 specimens of *Hydnellum* were collected with different morphological characteristics. The morphological observation and phylogenetic analyses based on ITS + nLSU and ITS + LSU + nSSU + RPB2 combined matrixes were conducted to confirm the affinity of the undescribed species corresponding to *Hydnellum*. Five new species were described in detail and illustrated.

## Materials and methods

### Morphological study

The specimens used in this study were deposited at the herbarium of the Institute of Microbiology, Beijing Forestry University (BJFC). Macro-morphological descriptions were based on field notes and laboratory measurements. The microscopic measures used in this study were followed by Sun et al. (2020, 2022) under a light microscope (Nikon Eclipse E 80i microscope, Nikon, Tokyo, Japan). Microscopic characteristics, measurements, and drawings were made from slide preparations stained with Cotton Blue and Melzer’s reagent, following Liu et al. (2021a). The following abbreviations were used: IKI = Melzer’s reagent, IKI– = neither amyloid or dextrinoid, KOH = 5% potassium hydroxide, CB = Cotton Blue, CB += cyanophilous, CB– = acyanophilous, L = mean spore length (arithmetic average of all spores), W = mean spore width (arithmetic average of all spores), Q = variation in the L/W rationes between the specimens studied, and n = number of spores measured from given number of specimens. A field Emission Scanning Electron Microscope (FESEM) Hitachi SU-8010 (Hitachi, Ltd., Tokyo, Japan) was used to photograph the ornamentation of the basidiospores, and the materials were studied at up to 2,200 times magnification, according to Song et al. (2021, 2022).

### DNA extraction, polymerase chain reaction amplification, and sequencing

The CTAB rapid plant genome extraction kit DN14 (Aidlab Biotechnologies, Beijing, China) was used to acquire total genomic DNA from dried specimens according to the manufacturer’s instructions with some modifications (Sun et al., 2022). ITS4 and ITS5 were used as primers for the internal transcribed spacer (ITS), LR0R and LR7 were used for the large subunit of nuclear ribosomal RNA gene (nLSU), NS1/NS4 were used for the small subunit of nuclear ribosomal RNA gene (nSSU), and 5F/7Cr were used to the second largest subunit of RNA polymerase II (RPB2) gene. The Polymerase Chain Reaction (PCR) procedure for ITS was as follows: initial denaturation at 95°C for 3 min, followed by 35 cycles at 94°C for 40 s, 56°C for 45 s, and 72°C for 1 min, and a final extension of 72°C for 10 min. The PCR procedure for nLSU and nSSU was as follows: initial denaturation at 94°C for 1 min, followed by 35 cycles at 94°C for 30 s, 50°C for 1 min, and 72°C for 1.5 min, and a final extension of 72°C for 10 min. The PCR process for RPB2 was as follows: initial denaturation at 94°C for 2 min, 9 cycles at 94°C for 45 s, 60°C for 45 s, followed by 36 cycles at 94°C for 45 s, 53°C for 1 min, 72°C for 90 s, and a final extension of 72°C for 10 min. The PCR products were purified and sequenced at the Beijing Genomics Institute, China, with the same primers. The newly generated sequences were deposited at GenBank. All sequences analyzed in this study were deposited at GenBank and are listed in Table 1.

**TABLE 1:** A list of species, specimens, and GenBank accession numbers of sequences used in this study.

Species	Specimen no.	Locality	GenBank accession no.				
			
			ITS	nrLSU	nSSU	RPB1	RPB2
*Amaurodon aquicoeruleus*	UK 452	Australia	AM490944	AM490944	-	-	-
*A. viridis*	KHLarsson14947b	Norway	MK602707	MK602707	-	-	-
*Hydnellum amygdaliolens*	GB-0202072	France	MW144290	MW144290	-	-	-
*H. amygdaliolens*	SC-2011	-	JN376763	-	-	-	-
*H. atrorubrum*	Wei 8315	China	MW579937	-	-	-	-
*H. atrorubrum*	Wei 8261	China	MW579936	MW579884	MW579910		
*H. atrospinosum*	Yuan 6514	China	MW579940	MW579886	MW579913	-	-
*H. atrospinosum*	Yuan 6520	China	MW579912	-	MW579912	-	-
*H. aurantiacum*	RGCarlsson08-105	Sweden	MK602711	MK602711	-	-	-
*H. aurantiacum*	EBendiksen177-07	Norway	MK602712	MK602712	-	-	-
*H. auratile*	OF294095	Norway	MK602714	MK602714	-	-	-
*H. auratile*	OF242763	Norway	MK602715	MK602715	-	-	-
*H. bomiense*	Yuan 13759	China	MW579941	MW579887	MW579914	-	OK254206
*H. bomiense*	Yuan 13767	China	MW579942	-	MW579915	-	-
*H. brunneorubrum*	Yuan 12997	China	MW579944	MW579889	MW579917	-	OK254217
*H. brunneorubrum*	Yuan 14339	China	MW57994 3	MW579888	MW579916	-	OK254216
*H. brunneorubrum*	Yuan 14668	China	MW57994 5	MW57989 0	MW579918	-	OK254218
*H. caeruleum*	OF291490	Norway	MK602717	MK602717	-	-	-
*H. caeruleum*	EBendiksen575-11	Norway	MK602718	MK602718	-	-	-
*H. chocolatum*	Cui 18545	China	**ON603657**	**-**	**-**	**-**	**ON605665**
*H. chocolatum*	Cui 18543	China	**ON603656**	**ON603638**	**ON603646**	**ON605658**	**-**
*H. chrysinum*	SC071	-	KJ534291	-	-	-	-
*H. coactum*	Wei 8094	China	MN846278	MN846287	-	-	-
*H. coactum*	Shi 181	China	MN846279	MN846288	-	-	-
*H. complicatum*	REB-71	United States	KC571711	-	-	-	-
*H. complicatum*	REB-329	United States	KC571712	-	-	-	-
*H. concentricum*	Cui 17017	China	**ON603658**	**ON603639**	**ON603647**	**ON605659**	**ON605666**
*H. concentricum*	Cui 17098	China	**-**	**ON603640**	**ON603648**	**ON605660**	**-**
*H. concrescens*	REB-385	United States	JN135182	-	-	-	-
*H. concrescens*	REB-65	United States	KC571713	-	-	-	-
*H. concrescens*	REB-384	United States	KC571714	-	-	-	-
*H. crassipileatum*	Cui 17021	China	**ON603660**	**ON603641**	**ON603649**	**ON605661**	**ON605668**
*H. crassipileatum*	Cui 17019	China	**ON603659**	**ON603642**	**ON603650**	**ON605662**	**-**
*H. cristatum*	4446	Canada	KM406974	-	-	-	-
*H. cristatum*	REB-169	United States	JN135174	-	-	-	-
*H. cumulatum*	SEW 69	United States	AY569026	-	-	-	-
*H. cumulatum*	REB-342	United States	JN135172	-	-	-	-
*H. cyanopodium*	SEW 85	United States	AY569027	-	-	-	-
*H. diabolus*	KAH13873	Canada	AF351863	-	-	-	-
*H. dianthifolium*	ML902162HY	-	KX619420	-	-	-	-
*H. dianthifolium*	ML61211HY	-	KX619419	-	-	-	-
*H. earlianum*	REB-375	United States	JN135179	-	-	-	-
*H. earlianum*	REB-75	United States	KC571724	-	-	-	-
*H. fagiscabrosum*	GB-0195621	Sweden	MW144293	MW144293	-	-	-
*H. fagiscabrosum*	GB-0195805	Sweden	MW144294	MW144294	-	-	-
*H. fagiscabrosum*	GB-0195625	Sweden	MW144292	MW144292	-	-	-
*H. fennicum*	OF242833	Norway	MK602738	MK602738	-	-	-
*H. fennicum*	SWesterberg110909	Sweden	MK602739	MK602739	-	-	-
*H. ferrugineum*	ELarsson 356-16	Sweden	MK602721	MK602721	-	-	-
*H. ferrugineum*	ELarsson 197-14	Sweden	MK602722	MK602722	-	-	-
*H. ferrugipes*	REB-176	United States	KC571727	-	-	-	-
*H. ferrugipes*	REB-68	United States	JN135176	-	-	-	-
*H. fibulatum*	Yuan 14646	China	MW579957	-	MW579926	-	-
*H. fibulatum*	Yuan 14656	China	MW579927	-	MW579958	-	-
*H. fuligineoviolaceum*	LA120818	Sweden	MK602740	MK602740	-	-	-
*H. fuligineoviolaceum*	BNylen130918	Sweden	MK602741	MK602741	-	-	-
*H. fuscoindicum*	OSC 113641	United States	EU669230	EU669280	-	-	-
*H. fuscoindicum*	OSC 107844	United States	EU669229	EU669279	-	-	-
*H. geogenium*	EBendiksen526-11	Norway	MK602725	MK602725	-	-	-
*H. geogenium*	OF296213	Norway	MK602724	MK602724	-	-	-
*H. geogenium*	OF66379	Norway	MK602723	MK602723	-	-	-
*H. glaucopus*	RGCarlsson13-060	Sweden	MK602743	MK602743	-	-	-
*H. glaucopus*	JNitare06091	Sweden	MK602744	MK602744	-	-	-
*H. gracilipes*	ELarsson 219-11	Sweden	MK602727	MK602727	-	-	-
*H. gracilipes*	GB-0113779	Sweden	MK602726	MK602726	-	-	-
*H. granulosum*	Yuan 12213a	China	MW579948	MW579893	MW579921	-	OK254213
*H. granulosum*	Yuan 12213b	China	MW579947	MW579892	MW579920	-	OK254212
*H. grosselepidotum*	Wei 8120	China	MN846274	MN846283	-	-	-
*H. grosselepidotum*	Wei 8015	China	MN846276	MN846285	-	-	-
*H. illudens*	O-F-76340	Norway	MW144334	MW144334	-	-	-
*H. illudens*	O-F-242769	Norway	MW144335	MW144335	-	-	-
*H. illudens*	O-F-68659	Norway	MW144333	MW144333	-	-	-
*H. inflatum*	Wang 80	China	MW579949	MW579949	MW579922	-	OK254210
*H. inflatum*	Shi 506	China	OK254210	MW579895	OK254210	-	OK254211
*H. joeides*	RGCarlsson11-090	Sweden	MK602749	MK602749	-	-	-
*H. joeides*	KHjortstam17589	Sweden	MK602750	MK602750	-	-	-
*H. joeides*	Nitare110829	Sweden	MK602751	MK602751	-	-	-
*H. lepidum*	EGrundel110916	Sweden	MK602753	MK602753	-	-	-
*H. lepidum*	JNitare110829	Sweden	MK602754	MK602754	-	-	-
*H. lidongensis*	Wei 8329	China	MN846281	MN846290	-	-	-
*H. lidongensis*	Wei 8365	China	MN846280	MN846289	-	-	-
*H. lundellii*	Stridvall06049	Sweden	MK602758	MK602758	-	-	-
*H. lundellii*	OF242639	Norway	MK602759	MK602759	-	-	-
*H. lundellii*	OF295814	Norway	MK602760	MK602760	-	-	-
*H. martioflavus*	OF242872	Norway	MK602761	MK602761	-	-	-
*H. martioflavus*	OF242435	Norway	MK602762	MK602762	-	-	-
*H. martioflavus*	ADelin110804	Sweden	MK602763	MK602763	-	-	-
*H. melanocarpum*	Cui 18556	China	**ON603661**	**-**	**ON603651**	**-**	**-**
*H. melanocarpum*	Cui 18557	China	**ON603662**	**ON603643**	**ON603652**	**-**	**-**
*H. melanocarpum*	Cui 18559	China	**ON603663**	**ON603644**	**ON603653**	**-**	**ON605667**
*H. mirabile*	SLund140912	Sweden	MK602730	MK602730	-	-	-
*H. mirabile*	RGCarlsson11-119	Sweden	MK602728	MK602728	-	-	-
*H. mirabile*	ELarsson170 14	Sweden	MK602729	MK602729	-	-	-
*H. nemorosum*	GB-0195631	Sweden	MW144373	MW144373	-	-	-
*H. nemorosum*	O-F-242352	Norway	MW144372	MW144372	-	-	-
*H. parvum*	REB-131	United States	JN135187	-	-	-	-
*H. parvum*	REB-392	United States	KC571717	-	-	-	-
*H. peckii*	SSvantesson328	Norway	MK602731	MK602731	-	-	-
*H. peckii*	ELarsson174-14	Sweden	MK602732	MK602732	-	-	-
*H. peckii*	EBendiksen 567-11	Norway	MK602733	MK602733	-	-	-
*H. pineticola*	REB-49	United States	KC571733	-	-	-	-
*H. pineticola*	REB-43	United States	JN135175	-	-	-	-
*H. piperatum*	REB-332	United States	JN135173	-	-	-	-
*H. piperatum*	REB-304	United States	KC571723	-	-	-	-
*H. radiatum*	Cui 17130	China	**ON603664**	**ON603645**	**ON603654**	**ON605663**	**ON605669**
*H. radiatum*	Cui 16254	China	**ON603665**	**-**	**ON603655**	**ON605664**	**-**
*H. regium*	SEW 93	United States	AY569031	-	-	-	-
*H. roseoviolaceum*	GB-0195687	Sweden	MW144375	MW144375	-	-	-
*H. roseoviolaceum*	GB-0195936	Sweden	MW144374	MW144374	-	-	-
*H. rubidofuscum*	Yuan 14587	China	MW579952	MW579897	MW579925	-	OK254208
*H. rubidofuscum*	Yuan 14561	China	MW579951	MW579896	MW579924	-	OK254207
*H. rubidofuscum*	Yuan 14654	China	MW579953	MW579898	-	-	OK254209
*H. scabrosellum*	GB-0195806	Sweden	MW144377	MW144377	-	-	-
*H. scabrosellum*	GB-0195791	Sweden	MW144378	MW144378	-	-	-
*H. scabrosellum*	GB-0195689	Sweden	MW144379	MW144379	-	-	-
*H. scabrosum*	OF295824	Norway	MK602764	MK602764	-	-	-
*H. scabrosum*	OF360777	Norway	MK602765	MK602765	-	-	-
*H. scabrosum*	OF292320	Norway	MK602766	MK602766	-	-	-
*H. scleropodium*	REB-3	United States	JN135186	-	-	-	-
*H. scleropodium*	REB-352	United States	KC571740	-	-	-	-
*H. scrobiculatum*	REB-78	United States	JN135181	-	-	-	-
*H. spongiosipes*	SEW 86	United States	AY569021	-	-	-	-
*H. spongiosipes*	REB-107	United States	KC571743	-	-	-	-
*H. spongiosipes*	REB-52	United States	JN135184	-	-	-	-
*H. squamulosum*	Yuan 13625	China	MW579956	MW579899	-	-	OK254204
*H. squamulosum*	Yuan 13743	China	MW579955	-	-	-	OK254203
*H. suaveolens*	ELarsson 139**-**09	Norway	MK602734	MK602734	-	-	-
*H. suaveolens*	ELarsson 8-14	Sweden	MK602735	MK602735	-	-	-
*H. suaveolens*	SSvantesson877	Norway	MK602736	-	-	-	-
*H. subsuccosum*	SEW 55	United States	AY569033	-	-	-	-
*H. subsuccosum*	REB-10	United States	JN135178	-	-	-	-
*H. sulcatum*	Yuan 14521	China	MW579961	MW579902	MW579930	-	OK254202
*H. sulcatum*	Yuan 14649	China	MW579960	MW579901	MW579929	-	OK254201
*H. sulcatum*	Yuan 14660	China	MW579959	MW579900	MW579901	-	-
*Hydnellum* sp.1	Shi 164	China	-	MW579969	-	-	-
*Hydnellum* sp.2	Yuan 14387	China	MW579970	MW579908	MW579934	-	-
*Hydnellum* sp.3	Yuan 14388	China	MW579971	-	-	-	-
*Hydnellum* sp.4	Wang 295	China	MW579972	-	-	-	-
*Hydnellum* sp.5	Yuan 14594	China	MW579973	MW579909	MW579935	-	OK254205
*H. yunnanense*	Yuan 14386	China	MW579962	MW579903	-	-	OK254199
*H. yunnanense*	Yuan 14396	China	MW579963	MW579904	-	-	OK254200
*H. underwoodii*	REB-358	United States	JN135189	-	-	-	-
*H. underwoodii*	REB-50	United States	KC571781	-	-	-	-
*H. versipellis*	EBendiksen164-07	Norway	MK602770	MK602770	-	-	-
*H. versipellis*	RGCarlsson13-057	Sweden	MK602771	MK602771	-	-	-
*Sarcodon aspratus*	-	-	DQ448877	-	-	-	-
*S. aspratus*	-	-	AF335110	-	-	-	-
*S. imbricatus*	JRova 1408292	Sweden	MK602746	MK602746	-	-	-
*S. imbricatus*	ELarsson 384-10	Norway	MK602747	MK602747	-	-	-
*S. imbricatus*	SSvantesson355	Norway	MK602748	MK602748	-	-	-
*S. scabripes*	REB-351	United States	JN135191	-	-	-	-
*S. scabripes*	FCME:23240	Mexico	EU293829	-	-	-	-
*S. squamosus*	ELarsson 248-12	Sweden	MK602767	MK602767		-	-
*S. squamosus*	OF177452	Norway	MK602768	MK602768	-	-	-
*S. squamosus*	OF295554	Norway	MK602769	MK602769	-	-	-
*S. quercinofibulatus*	JC-20090718.2	Italy	JX271818	MK602773	-	-	-
*S. quercinofibulatus*	TENN	United States	MG663244	-	-	-	-
*S. leucopus*	OF296099	Norway	MK602755	-	-	-	-
*S. leucopus*	OF296944	Norway	MK602756	-	-	-	-

New sequences are shown in bold.

**TABLE 2:** The distribution areas, ecological habits, and main morphological characters of species in *Hydnellum* from China.

Species	Distribution in China	Ecological habits	Alt.	Pileal surface (when fresh)	Spines color (when fresh)	Basidiospores (μm)	References
*H. atrorubrum*	Yunnan Province	on the ground of Fagaceous forest	2100 m	light brown to d arkruby	white to dark brown	(4.1–)4.5–6 × (3.2–)3.9–5.1(–6)	Mu et al., 2021
*H. atrospinosum*	Qinghai Province	on the ground of *Picea* forest	2800 m	light orange to yellowish brown	dark violet	(4–)4.1–5.1(–5.5) × (3–)3.1–3.9(–4)	Mu et al., 2021
*H. bomiense*	Xizang Autonomous Region	on the ground with moss of Fagaceous forest	2760 m	grayish yellow, brown to dark brown	white to brown	(4–)4.1–5.1(–5.2) × (3–)3.3–4.5(–4.8)	Mu et al., 2021
*H. brunneorubrum*	Liaoning Province	on the ground of Fagaceous forest or mixed forest	400 m	brownish orange to brownish red	golden yellow to light brown	(4–)4.1–5.1(–5.2) × (3.1–)3.2–4.6(–4.8)	Mu et al., 2021
*H. caeruleum*	Xinjiang Autonomous Region	on the ground of *Picea* forest	1900 m	pastel yellow to dark blonde	orange-white to dark brown	(4.9–)5–6(–6.1) × (4–)4.1–4.9(–5)	Mu et al., 2021
*H. chocolatum*	Sichuan Province	on the ground of mixed forest	3000 m	dark brown to fuscous	brown to grayish brown	4–5(–5.8) × (4.5–)5–6	This study
*H. coactum*	Yunnan Province	on the ground of Fagaceae forest	1600−2000 m	reddish-brown to dark brown	white to yellowish-white	(5.1–)5.7–7(–7.1) × (4.6–)4.7–5.9(–6)	Mu et al., 2020
*H. concentricum*	Yunnan Province	on the ground of forest dominated by trees of *Pinus* and *Quercus*	3000−3500 m	light brown, pastel red, reddish-brown to grayish brown	fawn to reddish brown	(3.2–) 3.5–5 × (3.5–)4–5(–5.2)	This study
*H. crassipileatum*	Yunnan Province	on the ground of forest dominated by trees of *Pinus* and *Quercus*	3525 m	reddish brown to grayish brown	grayish brown to fuscous	4–5.5 × 4–6 (–6.5)	This study
*H. fibulatum*	Liaoning Province	on the ground of *Quercus* forest	740 m	light brown to dark brown	pinkish white to brown	(4.2–)4.4–5.8(–6) × (4–)4.1–4.9(–5.1)	Mu et al., 2021
*H. granulosum*	Sichuan Province	on the ground of *Acer* and *Cryptomeria* mixed forest	1175 m	light yellow, light brown to grayish brown	grayish orange to dark brown when dry	(4–)4.1–5.1(–5.3) × (3.2–)3.4–4.7(–4.9)	Mu et al., 2021
*H. grosselepidotum*	Yunnan Province	on the ground of Fagaceae forest	2000 m	pale orange to dark ruby	white to pale yellow	(5–)5.1–6.4(–6.6) × (4–)4.1–5.9(–6)	Mu et al., 2020
*H. inflatum*	Yunnan Province	on the ground of Fagaceous forest	1580 m	grayish orange to brown	white to golden brown	(4–)4.2–5(–5.1) × (3.2–)3.8–4.3(–5)	Mu et al., 2021
*H. lidongensis*	Yunnan Province	on the ground of Fagaceous forest	2400 m	light brown to brown	grayish-orange to brown	(4–)4.1–6(–6.1) × (3.9–)4–5(–5.1)	Mu et al., 2020
*H. melanocarpum*	Sichuan Province	on the ground of mixed forest	4090 m	vinaceous brown	brown	(3.5–)3.8–5.1 × 4.5–5.5(–6)	This study
*H. peckii*	Xizang Autonomous Region	on the ground of *Pinus* mixed forest	2760 m	white to light orange	brownish orange	(4.1–)4.2–5.1(–5.3) × (3.8–)3.9–4.4(–4.6)	Mu et al., 2021
*H. radiatum*	Yunnan Province	on the ground of forest dominated by *Pinus armandii* and *Rhododendron* or *Pinus* and *Quercus*	2400−2700 m	dark brown, fuscous to black	dark brown	3–4.5(–5) × (3.5–) 4–5	This study
*H. rubidofuscum*	Liaoning Province	on the ground of *Quercus* forest	400 m	reddish brown	grayish brown to reddish brown	(4–)4.1–5(–5.1) × (3.8–)3.9–4.6(–4.8)	Mu et al., 2021
*H. spongiosipes*	Liaoning Province	on the ground of *Quercus* forest	400 m	pale orange to dark brown	pale orange to dark brown	(5–)5.1–6.1(–6.2) × (4.3–)4.5–5.3(–5.8)	Mu et al., 2021
*H. squamulosum*	Xizang Autonomous Region	on the ground with moss of *Picea* mixed forest	2760 m	pastel red to dark magenta	pale red to reddish brown	(4–)4.1–5(–5.1) × (3.2–)3.3–4.1(–4.2)	Mu et al., 2021
*H. sulcatum*	Liaoning Province	on the ground of *Quercus* forest	740 m	dark brown	brown	(4–)4.1–5.8(–5.9) × (3.9–)4–4.6(–4.8)	Mu et al., 2021
*H. yunnanense*	Yunnan Province	on the ground	2358 m	grayish red to dark brown	white to grayish red	(4.1–)4.2–5.1(–5.3) × (3.4–)3.5–4.5(–5)	Mu et al., 2021

### Phylogenetic analyses

The new sequences generated in this study were combined with the sequences downloaded from GenBank and are listed in Table 1. *Amaurodon aquicoeruleus* Agerer and *A. viridis* (Alb. and Schwein.) J. Schröt were used as the outgroups, according to Mu et al. (2021). Sequences were aligned by MAFFT v.7 with the G-INI-I option (Katoh and Standley, 2013) and manually adjusted in BioEdit v. 7.0.9. (Hall, 1999). Alignments were spliced in Mesquite v. 3.2. (Maddison and Maddison, 2017). The partition homogeneity test (PHT) (Farris et al., 1994) of the four-gene dataset was tested by PAUP v. 4.0b10 (Swofford, 2002) under 1,000 homogeneity replicates. The best-fit evolutionary model was selected with AIC (Akaike Information Criterion) using ModelTest 2.3 (Guindon and Gascuel, 2003; Darriba et al., 2012). Phylogenetic analyses were carried out according to the previous studies (Cui et al., 2019; Liu et al., 2022a).

Maximum parsimony (MP) analyses were applied to the combined datasets. The construction was performed in PAUP* version 4.0b10 (Swofford, 2002). All characters were equally weighted and gaps were treated as missing data. Trees were inferred using the heuristic search option with TBR branch swapping and 1,000 random sequence additions. Max trees were set to 5,000, branches of zero length were collapsed, and all parsimonious trees were saved. Clade robustness was assessed using a bootstrap analysis with 1,000 replicates (Felsenstein, 1985). Descriptive tree statistics of tree length (TL), consistency index (CI), retention index (RI), rescaled consistency index (RC), and homoplasy index (HI) were calculated for each maximum parsimonious tree generated. RAxML-HPC2 was used to construct the maximum likelihood (ML) analyses with the GTRGAMMA model. All model parameters were estimated by the program, and only the best ML tree from all searches was kept. The ML bootstrap values were performed using rapid bootstrapping with 1,000 replicates.

MrModeltest 2.3 (Posada and Crandall, 1998; Nylander, 2004) was used to determine the best-fit evolution model for each dataset for Bayesian inference (BI). BI was calculated using MrBayes 3.1.2 with four Markov chains running for two runs from random starting trees for one million generations, and trees were sampled every 100 generations (Ronquist and Huelsenbeck, 2003). The first 25% of the sampled trees were discarded as burn-in and a majority rule consensus tree of all remaining trees was calculated. All trees were viewed in FigTree v. 1.4.2.

## Results

### Phylogeny

**FIGURE 1:**
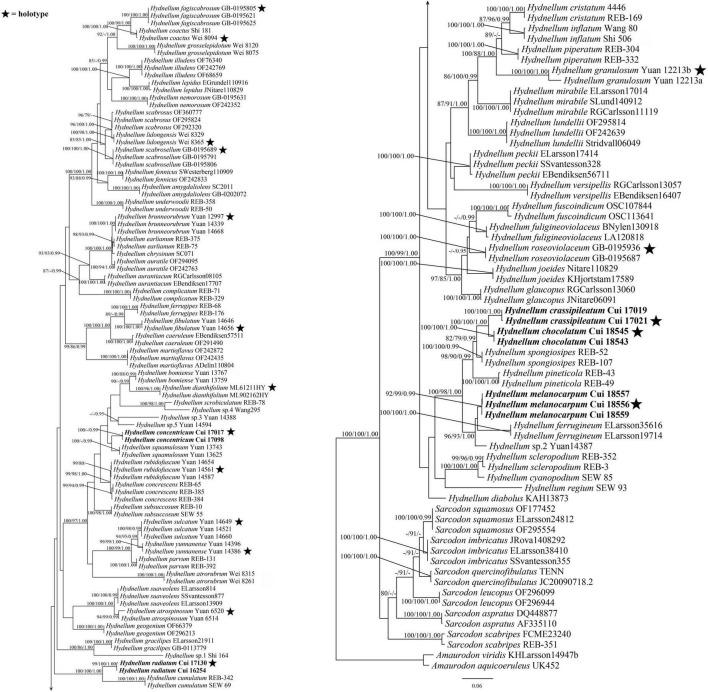
Maximum likelihood (ML) tree of the *Hydnellum* species based on ITS + nLSU sequences data. Branches are labeled with parsimony bootstrap values equal to or higher than 75%, maximum likelihood bootstrap values equal to or higher than 75%, and Bayesian posterior probabilities equal to or higher than 0.95. Bold names = New species.

The combined ITS + nLSU dataset included 257 sequences from 156 specimens representing 76 taxa. The dataset had an aligned length of 2,605 characters, including gaps (1,202 characters for ITS, 1,403 characters for nLSU), of which 1,527 characters were constant, 122 were variable and parsimony-uninformative, and 956 were parsimony-informative. Maximum parsimony analysis yielded 12 equally parsimonious trees (TL = 5,489, CI = 0.355, RI = 0.798, RC = 0.283, HI = 0.645). The best model for the combined ITS + nLSU sequences dataset estimated and applied in the Bayesian analysis was the GTR + I + G model. Bayesian and ML analysis resulted in a topology similar to that of MP analysis. Bayesian analysis has an average standard deviation of split frequencies = 0.005071. Only the ML tree was provided in Figure 1, and the MP bootstrap values (≥75%), ML bootstrap values (≥75%), and BPP (≥0.95) were shown at the nodes.

**FIGURE 2:**
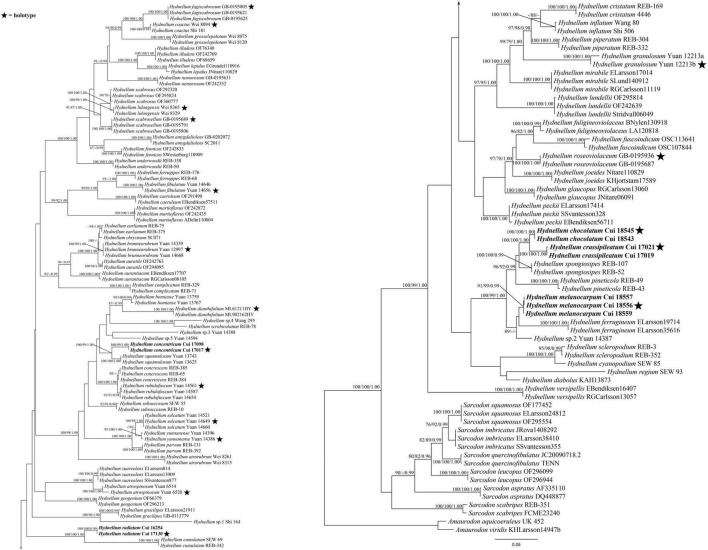
Maximum likelihood tree of the *Hydnellum* species based on the combined ITS + nLSU + nSSU + RPB2 sequences data. Branches are labeled with parsimony bootstrap values equal to or higher than 75%, ML bootstrap values equal to or higher than 75%, and Bayesian posterior probabilities equal to or higher than 0.95. Bold names = New species.

The combined ITS + nLSU + nSSU + RPB2 dataset included 312 sequences from 156 specimens representing 76 taxa. The dataset had an aligned length of 4,639 characters, including gaps (1,181 characters for ITS, 1,399 characters for nLSU, 986 characters for nSSU, and 1,073 characters for RPB2), of which 3,168 characters were constant, 186 were variable and parsimony-uninformative, and 1,285 were parsimony-informative. Maximum parsimony analysis yielded 15 equally parsimonious trees (TL = 6,171, CI = 0.392, RI = 0.800, RC = 0.313, HI = 0.608). The best model for the combined ITS + nLSU + nSSU + RPB2 sequences dataset estimated and applied in the Bayesian analysis was the GTR + I + G model. Bayesian and ML analysis resulted in a topology similar to that of MP analysis. Bayesian analysis has an average standard deviation of split frequencies = 0.007966. Only the ML tree was provided in Figure 2, and the MP bootstrap values (≥75%), ML bootstrap values (≥75%), and BPP (≥0.95) were shown at the nodes.

### Taxonomy

**FIGURE 3:**
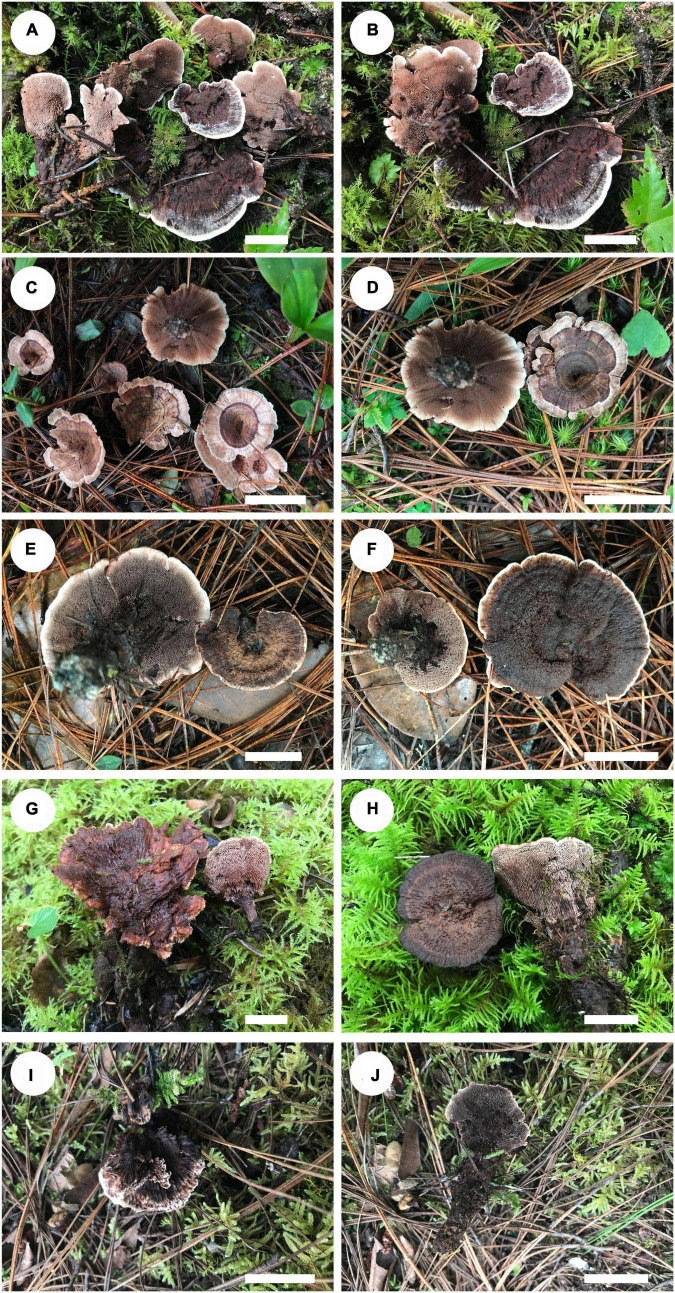
Basidiomata of *Hydnellum* species. **(A)** *H. chocolatum* (paratype, Cui 18543), **(B)** *H. chocolatum* (holotype Cui 18545), **(C,D)** *H. concentricum* (holotype, Cui 17017), **(E,F)** *H. crassipileatum* (paratype, Cui 17019), **(G)** *H. melanocarpum* (paratype, Cui 18557), and **(H)** *H. melanocarpum* (paratype, Cui 18559), **(I,J)** *H. radiatum* (holotype, Cui 17130) Scale bars: 2 cm.

**FIGURE 4:**
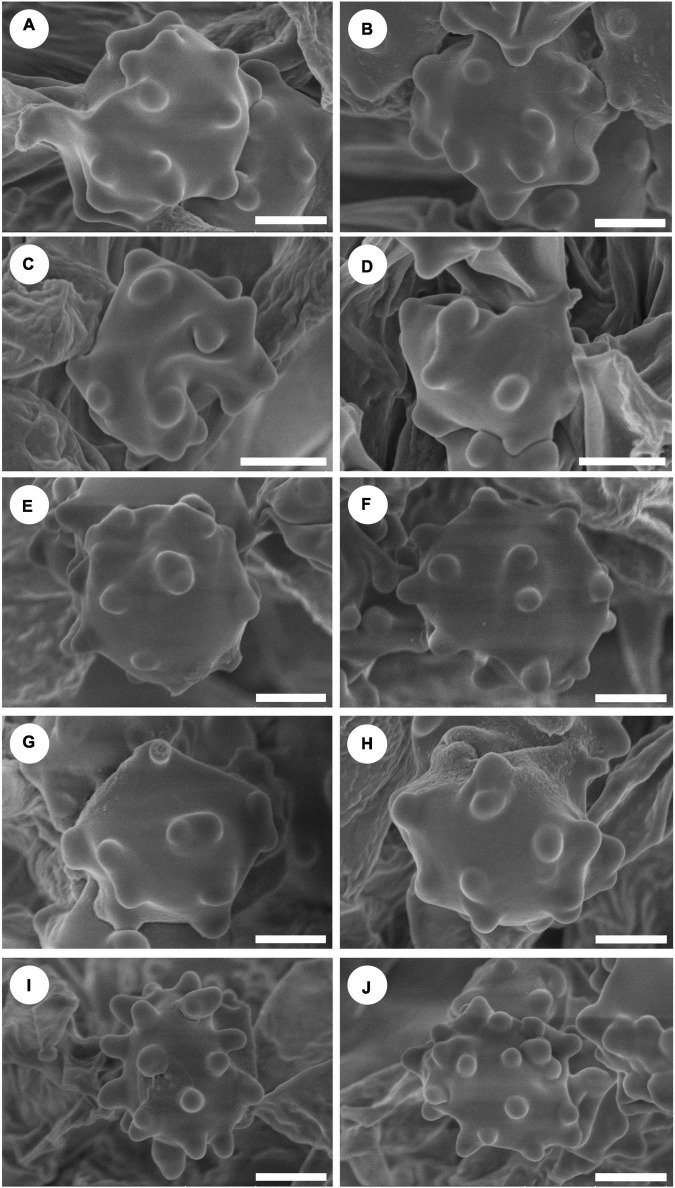
Scanning Electron Microscope (SEM) of basidiospores of *Hydnellum* species. **(A,B)** *H. chocolatum*, **(C,D)** *H. concentricum*, **(E,F)** *H. crassipileatum*, **(G,H)** *H. melanocarpum*, and **(I,J)** *H. radiatum* Scale bars: 1.5 μm.

**FIGURE 5:**
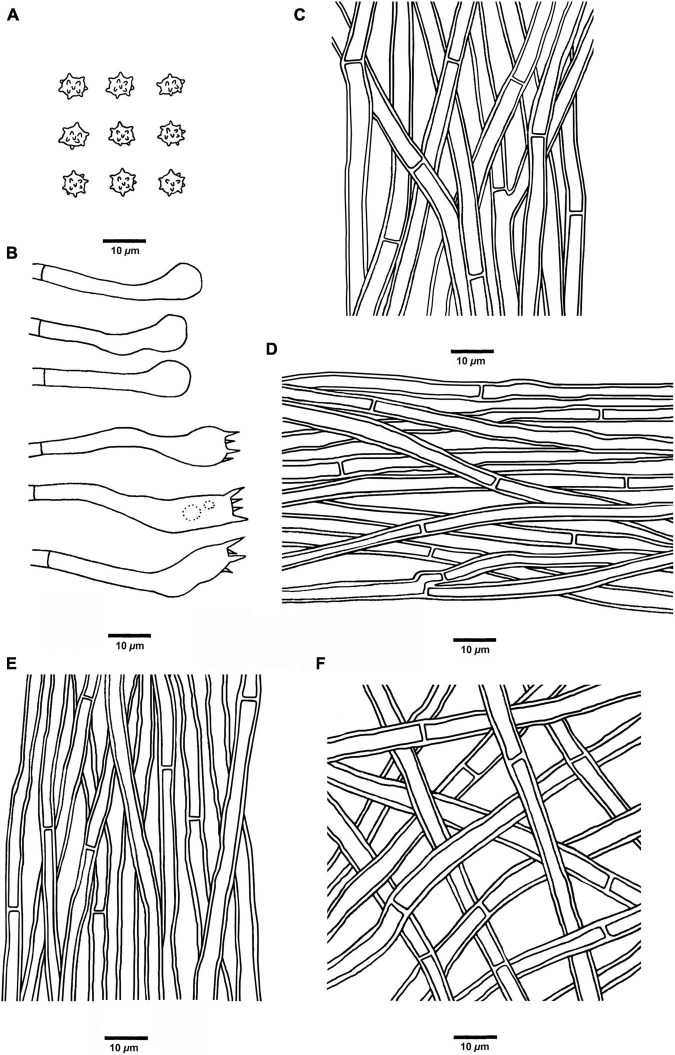
Microscopic structures of *H. chocolatum* (drawn from Cui 18545). **(A)** Basidiospores, **(B)** Basidia and basidioles, **(C)** Hyphae from context, **(D)** Hyphae from spines, **(E)** Hyphae from the inner layer of stipe, and **(F)** Hyphae from the surface layer of stipe.

***Hydnellum chocolatum*** B. K. cui and C. G. Song, sp. nov. (Figures 3A,B, 4A,B, 5).

MycoBank no.: 846115

*Diagnosis:* Differs from others by its fibrillose, spongy to tomentose pileal surface in chocolate color.

*Type:* CHINA. Sichuan Province, Jiuzhaigou County, on the ground of the mixed forest, elev. 2,600 m, 19 September 2020, Bao-Kai Cui, Cui 18545 (holotype, BJFC 035406).

*Etymology: chocolatum* (Lat.) refers to the chocolate-colored pileal surface.

*Fruiting body:* Basidiomata annual, eccentrically stipitate, single to concrescent, and odorless when fresh. The pileus is circular to irregular, with irregular folds in the middle, and up to 7.4 cm in diam and 0.7 cm thick at the center. Pileal surface is chocolate to fuscous when fresh, becoming grayish brown upon drying, azonate, fibrillose to spongy at the center, and tomentose near the margin, with radially aligned stripes toward the margin; margin white to light brown when fresh, becoming grayish brown upon drying, up to 7 mm wide. Context is brown to vinaceous gray upon drying, corky to fragile, and up to 3 mm thick. Spines are soft, brown to grayish brown when fresh, becoming grayish brown upon drying, fragile, and up to 5 mm long. Stipe is cylindrical and glabrous, surface layer is dark brown to vinaceous gray, and inner layer is grayish brown, and up to 3.8 cm long and 0.9 cm in diam.

*Hyphal structure:* Hyphal system monomitic; generative hyphae with simple septa; all the hyphae IKI–, CB–; tissues turned to olive-green or black in KOH.

*Context:* Generative hyphae grayish brown, thick-walled, branched, regular arranged, 2.5 to 6 μm in diam.

*Spines:* Generative hyphae clay-buff, slightly thick-walled, occasionally branched, regular arranged, 2 to 4.5 μm in diam. Cystidia and cystidioles are absent. Basidia clavate, bearing four sterigmata (2–4 μm long) and a basal simple septum, 32–45 × 5–7 μm; basidioles similar to basidia in shape, but slightly smaller.

*Stipe:* Generative hyphae clay-buff to grayish brown, slightly thick-walled, rarely branched, interwoven in the surface layer, regularly arranged in the inner layer, and 2 to 4 μm in diam.

*Spores:* Basidiospores subglobose to globose, hyaline, thin-walled, echinulate, IKI–, CB– (4.5–)5–6 × 4–5(–5.8) μm, *L* = 5.2 μm, *W* = 4.7 μm, *Q* = 1–1.25 (*n* = 60/2, without the ornamentation).

*Additional specimen (paratype) examined:* **CHINA**. Sichuan Province, Jiuzhaigou County, on the ground of the mixed forest, elev. 2,600 m, 19 September 2020, Bao-Kai Cui, Cui 18543 (BJFC 035404).

*Ecological habits: Hydnellum chocolatum* was collected in Southwest China under a plateau humid climate. It grows on the moist ground of the mixed forest, with well-watered bryophytes.

**FIGURE 6:**
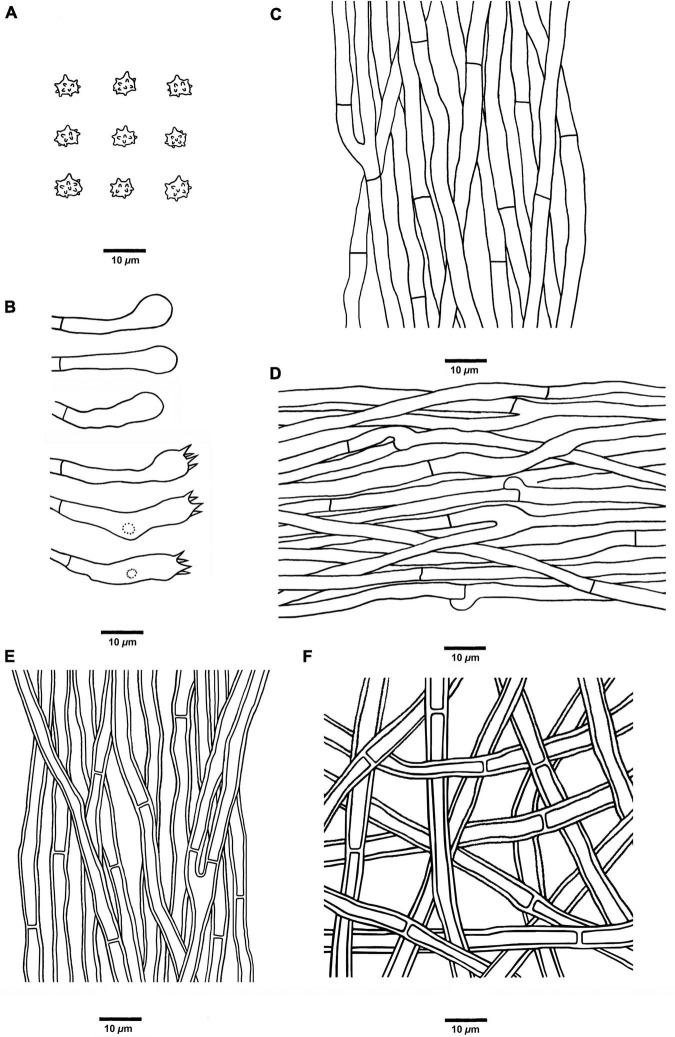
Microscopic structures of *H. concentricum* (drawn from Cui 17017). **(A)** Basidiospores, **(B)** Basidia and basidioles, **(C)** Hyphae from context, **(D)** Hyphae from spines, **(E)** Hyphae from the inner layer of stipe, and **(F)** Hyphae from the surface layer of stipe.

***Hydnellum concentricum*** B. K. Cui and C. G. Song, sp. nov. (Figures 3C,D, 4C,D, 6).

MycoBank no.: 846116

*Diagnosis:* Differs from other *Hydnellum* species by its zonate pileal surface, thin context, short stipe, and presence of both simple septa and clamp connections in generative hyphae of spines.

*Type:* CHINA. Yunnan Province, Lijiang City, Yulong County, Jiuhe, Laojun Mountain, Jiushijiulongtan, on the ground of forest dominated by trees of *Pinus* sp. and *Quercus* sp., elev. 2,800 m, 15 September 2018, Bao-Kai Cui, Cui 17017 (holotype, BJFC 030316).

*Etymology: concentricum* (Lat.) refers to the concentric bands on the pileal surface.

*Fruiting body:* Basidiomata annual, centrally stipitate, single, and odorless when fresh. Pileus infundibuliform, and up to 3.2 cm in diam and 0.4 cm thick at the center. Pileal surface is light brown, pastel red, reddish-brown to grayish brown when fresh, becoming brown to grayish brown upon drying, zonate, glabrous, with radially aligned stripes; margin fawn to orange-brown when fresh, becoming fawn upon drying, and up to 0.8 cm wide. Context is grayish brown upon drying, fragile, and up to 1 mm thick. Spines are soft, fawn to reddish-brown when fresh, grayish brown upon drying, fragile, and up to 3 mm long. Stipe cylindrical, glabrous, surface layer honey yellow to grayish brown upon drying, inner layer grayish brown upon drying; and up to 1.8 cm long and 0.5 cm in diam.

*Hyphal structure:* Hyphal system monomitic; generative hyphae in context and stipe with simple septa, generative hyphae in spines mostly with simple septa, occasionally with clamp connections; all the hyphae IKI–, CB–; tissues turned to olive-green or black in KOH.

*Context:* Generative hyphae clay-buff to grayish brown, slightly thick-walled, branched, regularly arranged, and 2 to 6 μm in diam.

*Spines:* Generative hyphae clay-buff, thin-walled, occasionally branched, occasionally with clamp connections, regularly arranged, and 2 to 4.5 μm in diam. Cystidia and cystidioles are absent. Basidia clavate, bearing four sterigmata (2–4 μm long) and a basal simple septum, 22–48 × 5–7 μm; basidioles are similar to basidia in shape but slightly smaller.

*Stipe:* Generative hyphae clay-buff, slightly thick-walled, rarely branched, interwoven in the surface layer, regularly arranged in the inner layer, and 2 to 4.5 μm in diam.

*Spores:* Basidiospores subglobose to ellipsoidal, hyaline, thin-walled, echinulate, IKI–, CB– (3.5–)4–5(–5.2) × (3.2–)3.5–5 μm, *L* = 4.6 μm, *W* = 3.9 μm, *Q* = 1.04–1.37 (*n* = 60/2, without the ornamentation).

*Additional specimen (paratype) examined:* **CHINA**. Yunnan Province, Shangri-La, on the ground of forest dominated by trees of *Pinus yunnanensis*, elev. 3,200 m, 17 September 2018, Bao-Kai Cui, Cui 17098 (BJFC 030398).

*Ecological habits: Hydnellum concentricum* was collected in Southwest China, under a plateau monsoon climate. It grows on the moist ground of a forest dominated by trees of *Pinus yunnanensis*.

**FIGURE 7:**
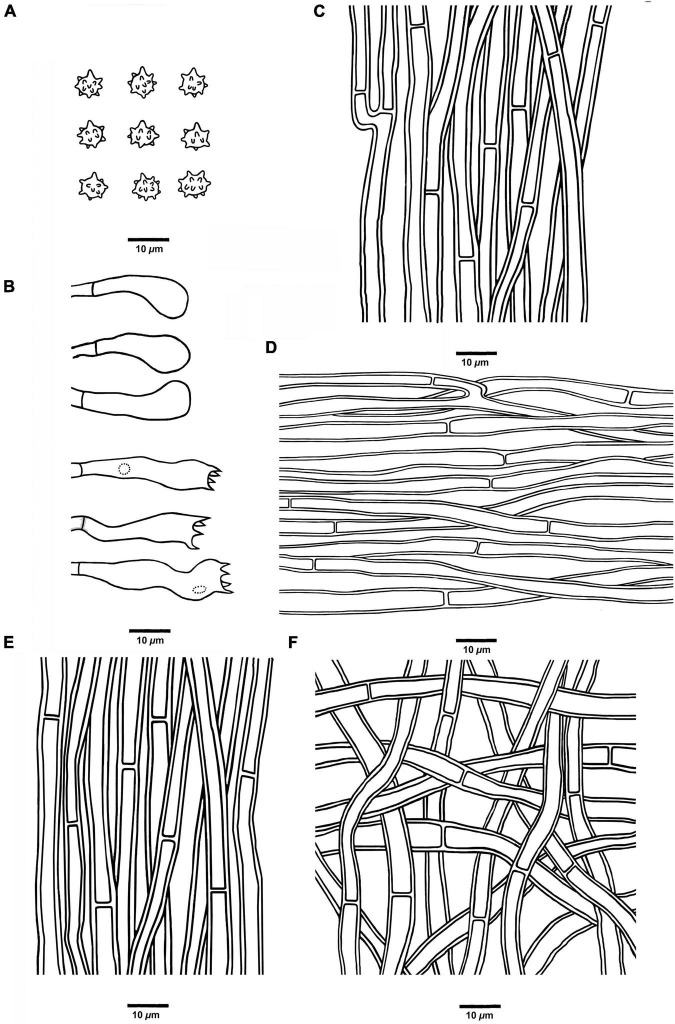
Microscopic structures of *H. crassipileatum* (drawn from Cui 17021). **(A)** Basidiospores, **(B)** Basidia and basidioles, **(C)** Hyphae from context, **(D)** Hyphae from spines, **(E)** Hyphae from the inner layer of stipe, and **(F)** Hyphae from the surface layer of stipe.

***Hydnellum crassipileatum*** B. K. Cui and C. G. Song, sp. nov. (Figures 3E,F, 4E,F, 7).

MycoBank no.: 846117

*Diagnosis:* Differs from other *Hydnellum* species by its thick pileus with reddish brown to grayish brown pileal surface.

*Type:* CHINA. Yunnan Province, Lijiang City, Yulong County, Jiuhe, Laojun Mountain, Jiushijiulongtan, on the ground of forest dominated by trees of *Pinus* sp. and *Quercus* sp., elev. 2,800 m, 15 September 2018, Bao-Kai Cui, Cui 17021 (holotype, BJFC 030320).

*Etymology: crassipileatum* (Lat.) refers to the thick pileus.

*Fruiting body:* Basidiomata annual, eccentrically stipitate, single, and odorless when fresh. Pileus circular to elliptical, up to 5.5 cm in diam, and 0.5 cm thick at the center. Pileal surface is reddish-brown to grayish brown when fresh, becoming grayish brown upon drying, azonate, fibrillose, spongy to tomentose when young, and glabrous with age; margin white to grayish brown when fresh, becoming olivaceous buff upon drying, up to 6 mm wide. Context light grayish-brown upon drying, fragile, up to 5 mm thick. Spines soft, grayish brown to fuscous when fresh, fuscous to black upon drying, fragile, up to 4 mm long. Stipe cylindrical, glabrous, surface layer grayish-brown, inner layer grayish brown to fuscous; up to 4.8 cm long, and 1.9 cm in diam.

*Hyphal structure:* Hyphal system monomitic; generative hyphae with simple septa; all the hyphae IKI–, CB–; tissues turned to olive-green or black in KOH.

*Context:* Generative hyphae clay-buff, thick-walled, occasionally branched, regularly arranged, and 2.5 to 5 μm in diam.

*Spines:* Generative hyphae clay-buff, thick-walled, occasionally branched, more or less regularly arranged, and 2.5 to 4 μm in diam. Cystidia and cystidioles are absent. Basidia clavate, bearing four sterigmata (2.5–3.5 μm long) and a basal simple septum, 14–31 × 5–7 μm; basidioles similar to basidia in shape, but slightly smaller.

*Stipe:* Generative hyphae clay-buff, slightly thick-walled, rarely branched, interwoven in the surface layer, regularly arranged in the inner layer, and 2.5 to 5 μm in diam.

*Spores:* Basidiospores subglobose to ellipsoidal, hyaline, thin-walled, echinulate, IKI–, CB–, 4–6(–6.5) × 4–5.5 μm, *L* = 5.6 μm, *W* = 4.5 μm, *Q* = 1–1.38 (*n* = 60/2, without the ornamentation).

*Additional specimen (paratype) examined:* **CHINA**. Yunnan Province, Lijiang City, Yulong County, Jiuhe, Laojun Mountain, Jiushijiulongtan, on the ground of forest dominated by trees of *Pinus* and *Quercus*, elev. 2,800 m, 15 September 2018, Bao-Kai Cui, Cui 17019 (BJFC 030318).

*Ecological habits: Hydnellum crassipileatum* was collected in Southwest China, under a plateau monsoon climate. It grows on the ground of a moist forest dominated by trees of *Pinus* and *Quercus*.

**FIGURE 8:**
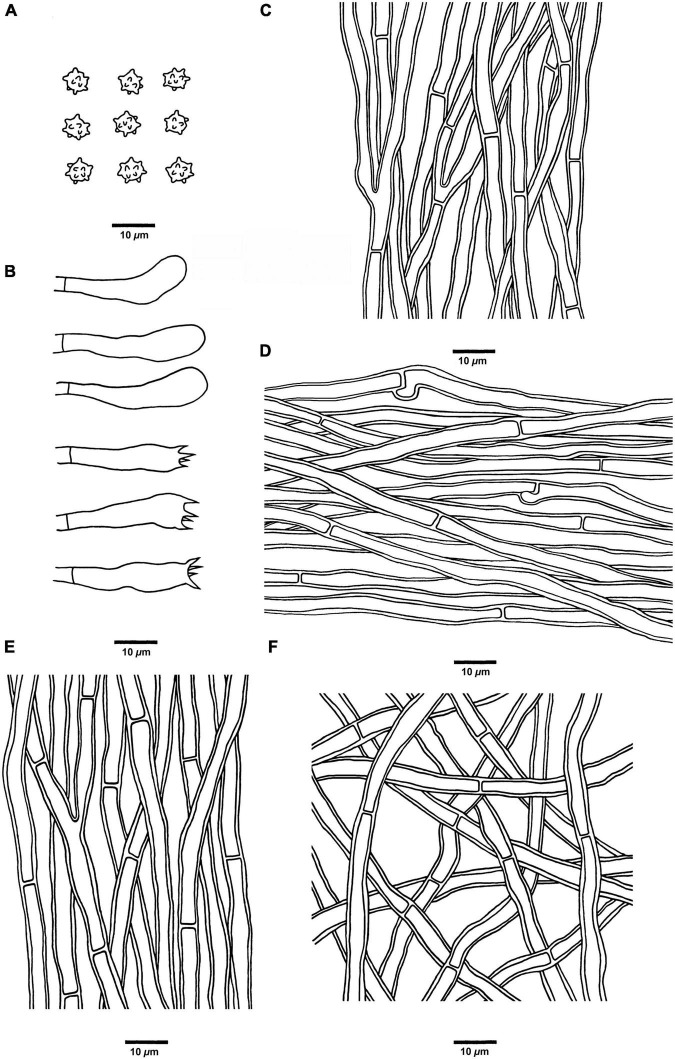
Microscopic structures of *H. melanocarpum* (drawn from Cui 18556). **(A)** Basidiospores, **(B)** Basidia and basidioles, **(C)** Hyphae from context, **(D)** Hyphae from spines, **(E)** Hyphae from the inner layer of stipe, and **(F)** Hyphae from the surface layer of stipe.

***Hydnellum melanocarpum*** B. K. Cui and C. G. Song, sp. nov. (Figures 3G,H, 4G,H, 8).

MycoBank no.: 846118

*Diagnosis:* Differs from other *Hydnellum* species by its vinaceous brown to black pileus with spongy pileal surface, and the presence of both simple septa and clamp connections in generative hyphae of spines.

*Type:* CHINA. Sichuan Province, Jiuzhaigou County, Jiuzhaigou Reverse, on the ground of the mixed forest, elev. 2,500 m, 20 September 2020, Bao-Kai Cui, Cui 18556 (holotype, BJFC 035417).

*Etymology: melanocarpum* (Lat.) refers to the vinaceous brown to black pileus.

*Fruiting body:* Basidiomata annual, centrally or eccentrically stipitate, single to concrescent, and odorless when fresh. Pileus is circular to irregular, up to 4.8 cm in diam, and 0.7 cm thick at the center. Pileal surface is vinaceous brown to black when fresh and becoming grayish brown upon drying, azonate, and glabrous to spongy at the center; margin cream, clay-buff, to orange-brown when fresh, light vinaceous gray at the lower tip, and becoming grayish brown to fuscous upon drying, and up to 0.6 cm wide. Spines are soft, brown when fresh, grayish brown to black upon drying, fragile, and up to 4 mm long. Context is grayish brown upon drying, fragile, and up to 3 mm thick. Stipe is cylindrical, glabrous, and grayish brown; and up to 2.6 cm long and 0.8 cm in diam.

*Hyphal structure:* Hyphal system monomitic; generative hyphae in context and stipe with simple septa, generative hyphae in spines mostly with simple septa, occasionally with clamp connections; all the hyphae IKI–, CB–; tissues turned to olive green in KOH.

*Context:* Generative hyphae clay-buff to grayish brown, thick-walled, branched, regularly arranged, and 2 to 4 μm in diam.

*Spines:* Generative hyphae clay-buff, thin-walled, occasionally branched, regularly arranged, and 2 to 3.5 μm in diam. Cystidia and cystidioles are absent. Basidia clavate, bearing four sterigmata (1.5–3 μm long) and a basal simple septum, 18–38 × 5–7 μm; basidioles similar to basidia in shape, but slightly smaller.

*Stipe:* Generative hyphae clay-buff, slightly thick-walled, rarely branched, interwoven in the surface layer, regularly arranged in the inner layer, and 2 to 4 μm in diam.

*Spores:* Basidiospores subglobose, hyaline, thin-walled, echinulate, IKI–, CB–, 4.5–5.5(–6) × (3.5–)3.8–5.1 μm, *L* = 5 μm, *W* = 4.6 μm, *Q* = 1–1.25 (*n* = 90/3, without the ornamentation).

*Additional specimens (paratypes) examined:* **CHINA**. Sichuan Province, Jiuzhaigou County, Jiuzhaigou Nature Reverse, on the ground of the mixed forest, elev. 2,500 m, 20 September 2020, Bao-Kai Cui, Cui 18557 (BJFC 035418) and Cui 18559 (BJFC 035420).

*Ecological habits: Hydnellum melanocarpum* was collected in Southwest China, under a plateau monsoon climate. It grows on the ground of the mixed forest, in well-watered bryophytes, and its roots are often interspersed with pine needles.

**FIGURE 9:**
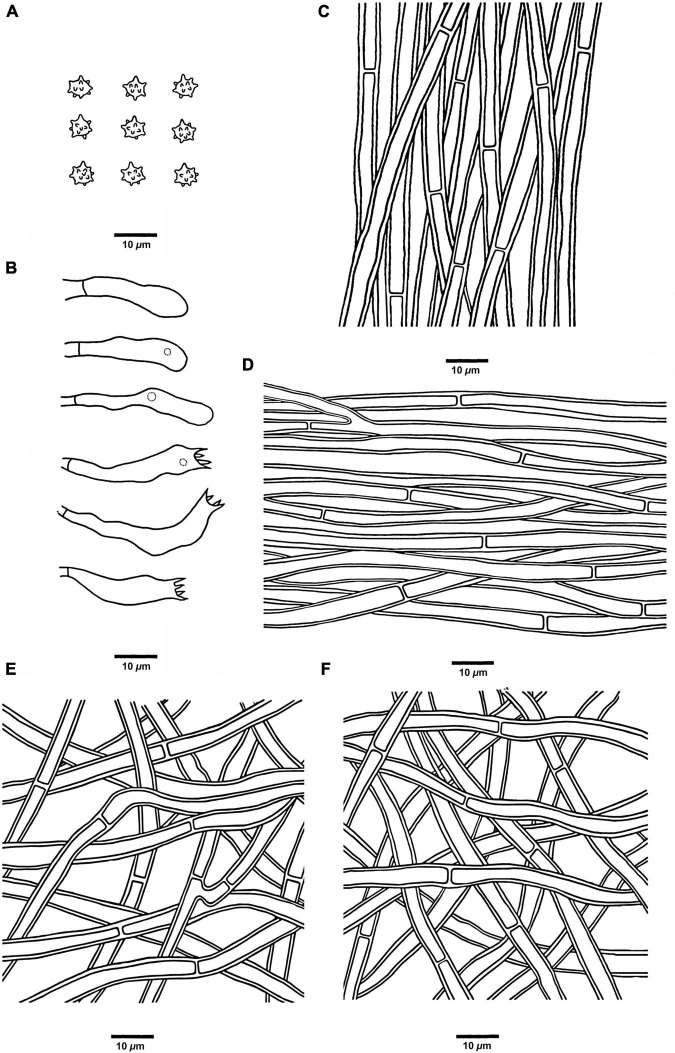
Microscopic structures of *H. radiatum* (drawn from Cui 17130). **(A)** Basidiospores, **(B)** Basidia and basidioles, **(C)** Hyphae from context, **(D)** Hyphae from spines, **(E)** Hyphae from the inner layer of stipe, and **(F)** Hyphae from the surface layer of stipe.

***Hydnellum radiatum*** B. K. Cui and C. G. Song, sp. nov. (Figures 3I,J, 4I,J, 9).

MycoBank no.: 846120

*Diagnosis:* Differs from other *Hydnellum* species by its radially aligned stripes on pileal surface, grayish brown context, and short stipe.

*Type:* CHINA. Yunnan Province, Lanping County, Tongdian, Jianganchang, on the ground of forest dominated by *Pinus armandii* and Rhododendron, elev. 2,480 m, 18 September 2018, Bao-Kai Cui, Cui 17130 (holotype, BJFC 030430).

*Etymology: radiatum* (Lat.) refers to the radially aligned stripes on the pileal surface.

*Fruiting body:* Basidiomata annual, eccentrically stipitate, single, and odorless when fresh. Pileus is subcircular, plicate, and up to 2.9 cm in diam and 0.5 cm thick at the center. Pileal surface is dark brown, fuscous to black when fresh and becoming fuscous to black upon drying, azonate, fibrillose, and with strong radially aligned stripes; margin white, cream to light brown when fresh, becoming grayish brown upon drying, and up to 0.3 cm wide. Context is grayish brown upon drying, tough, and up to 3 mm thick. Spines are soft, fuscous to black when fresh, grayish brown upon drying, fragile, and up to 3 mm long. Stipe is cylindrical, glabrous, surface layer fuscous to black upon drying, inner layer grayish brown upon drying, and up to 4 cm long and 1 cm in diam.

*Hyphal structure:* Hyphal system monomitic; generative hyphae with simple septa; all the hyphae IKI–, CB–; tissues of pileus and spines turned to olive green in KOH, tissues of stipe without reaction.

*Context:* Generative hyphae clay-buff, thick-walled, branched, regularly arranged, and 2 to 5.5 μm in diam.

*Spines:* Generative hyphae clay-buff, thick-walled, occasionally branched, regularly arranged, and 2 to 3.5 μm in diam. Cystidia and cystidioles are absent. Basidia clavate, bearing four sterigmata (1.5–4 μm long) and a basal simple septum, 14–21 × 3–4 μm; basidioles similar to basidia in shape, but slightly smaller.

*Stipe:* Generative hyphae clay-buff, thick-walled, rarely branched, interwoven in both the surface layer and the inner layer, and 2 to 5 μm in diam.

*Spores:* Basidiospores subglobose to ellipsoidal, hyaline, thin-walled, echinulate, IKI–, CB– (3.5–)4–5 × 3–4.5(–5) μm, *L* = 4.5 μm, *W* = 3.8 μm, *Q* = 1–1.35 (*n* = 60/2, without the ornamentation).

*Additional specimen (paratype) examined:* **CHINA**. Yunnan Province, Lanping County, Tongdian, Luoguqing, on the ground of forest dominated by *Pinus* and *Quercus*, elev. 2,630 m, 18 September 2017, Bao-Kai Cui, Cui 16254 (BJFC 029553).

*Ecological habits: Hydnellum radiatum* was collected in Southwest China, under a plateau monsoon climate. It grows on the ground of the forest dominated by trees of *Pinus yunnanensis*, in well-watered bryophytes, and its roots are often interspersed with pine needles.

Key to species of *Hydnellum* from China:

(1) Pileal surface scaled………………………………………………………… 2
(1) Pileal surface not scaled………………………………………………….. 3
(2) Pileal surface pale orange to dark ruby…………………………………………………………………………………….. *H. grosselepidotum*
(2) Pileal surface differently colored………………………………………………………………………………………………………….. *H. lidongensis*
(3) Pileus subinfundibuliform to infundibuliform………………. 4
(3) Pileus differently shaped……………………………………………….. 8
(4) Pileal surface glabrous……………………………. *H. concentricum*
(4) Pileal surface not glabrous……………………………………………… 5
(5) Pileal surface brownish orange to brownish red……………………………………………………………………….. *H. brunneorubrum*
(5) Pileus surface differently colored……………………………………. 6
(6) Pileal surface light brown to dark ruby………………………………………………………………………………………………. *H. atrorubrum*
(6) Pileus surface differently colored…………………………………… 7
(7) Pileus and spines grayish red…………………………………………………………………………………………………….. *H. yunnanense*
(7) Pileus and spines differently colored………………………………. …………………………………………………………………….. *H. bomiense*
(8) Context tissue becoming blue-green in KOH………………………………………………………………………………………….. *H. peckii*
(8) Context tissue differently colored in KOH……………………… 9
(9) Spines dark violet…………………………………… *H. atrospinosum*
(9) Spines differently colored…………………………………………….. 10
(10) Clamp connections present………………………………………….. 11
(10) Clamp connections absent…………………………………………… 13
(11) Pileal surface pastel yellow to dark blonde…………………………………………………………………………………….. *H. caeruleum*
(11) Pileal surface differently colored………………………………….. 12
(12) Clamp connections present in spines………………………………. …………………………………………………………….. *H. melanocarpum*
(12) Clamp connections absent in spines…………………………………………………………………………………………………… *H. fibulatum*
(13) Pileal surface zonate……………………………………………………… 14
(13) Pileal surface azonate…………………………………………………… 16
(14) Pileal surface pastel red to dark magenta………………………………………………………………………………….. *H. squamulosum*
(14) Pileal surface differently colored…………………………………… 15
(15) Pileal surface glabrous to scrupose when fresh……………………………………………………………………. *H. rubidofuscum*
(15) Pileal surface scabrous to fibrous when fresh……………………………………………………………………………….. *H. sulcatum*
(16) Pileal surface with radially aligned stripes………………………………………………………………………………………………………… 17
(16) Pileal surface without radially aligned stripes…………………………………………………………………………………………………… 18
(17) Spines fuscous to black…………………………………………………………………………………………………………………………. *H. radiatum*
(17) Spines brown to grayish brown……………………………………………………………………………………………………. *H. chocolatum*
(18) Spines white to yellowish-white………………………. *H. coatum*
(18) Spines differently colored…………………………………………. 19
(19) Context grayish orange……………………………………………………………………………………………………………………… *H. granulosum*
(19) Context differently colored…………………………………………. 20
(20) Pileal surface reddish brown to grayish brown……………………………………………………………………………. *H. crassipileatum*
(20) Pileal surface differently colored…………………………………. 21
(21) Stipe light brown………………………………………….. *H. inflatum*
(21) Stipe orange white, pale orange, sunburn to cognac…………………………………………………………………. *H. spongiosipes*

## Discussion

The genus *Hydnellum* is easy to recognize in Bankeraceae by its corky to woody pileus with crowded spines, but identification among the species in *Hydnellum* is difficult due to the quite similar morphological features. The main morphological characters of each species in *Hydnellum* from China were summarized in Table 2. The morphological differences between five new species were emphasized here briefly.

*Hydnellum chocolatum* was clustered with *H. crassipileatum* and *H. spongiosipes* (Peck) Pouzar in our phylogenetic analyses (Figures 1, 2). Morphologically, *H. chocolatum* resembles *H. crassipileatum* and *H. spongiosipes* in having single to concrescent basidiomata. However, *H. chocolatum* can be distinguished by its tomentose and azonate pileal margin, and longer basidia (32–45 × 5–7 μm); *H. crassipileatum* differs by its thick pileus with the reddish brown to grayish brown pileal surface; *Hydnellum spongiosipes* can be distinguished by its orange white to pale orange pileus and longer basidiospores [6–7 × 5–6 μm in *H. spongiosipes* vs. (4.5–)5–6 × 4–5(–5.8) μm in *H. chocolatum*, Baird et al., 2013].

Our phylogenetic analyses showed that *Hydnellum concentricum* was sister to *H. squamulosum* Y. H. Mu and H. S. Yuan (Figures 1, 2). The two species were both described in Southwest China, and share the annual, solitary to gregarious basidiomata, pastel red pileus, and reddish-brown spines (Mu et al., 2021). However, *H. squamulosum* differs from *H. concentricum* by its floccose to woolly, squamulose pileal surface and smaller basidiospores [4.1–5 × 3.3–4.1 μm in *H. squamulosum* vs. (3.5–)4–5(–5.2) × (3.2–)3.5–5 μm in *H. concentricum*, Mu et al., 2021].

*Hydnellum melanocarpum* is closely related to *Hydnellum ferrugineum* (Fr.) P. Karst. In our phylogenetic analyses (Figures 1, 2). However, *H. ferrugineum* differs from *H. melanocarpum* by its pale orange to burnt umber pileus, and larger basidiospores [5–6(–7) × 5–6 μm in *H. ferrugineum* vs. 4.5–5.5(–6) × (3.5–)3.8–5.1 μm *H. melanocarpum*, Baird et al., 2013].

*Hydnellum radiatum* resembles *H. cumulatum* in having similar colored spines, which have close phylogenetic relationship (Figures 1, 2). However, *H. cumulatum* can be distinguished by its vinaceous buff, hessian brown to burnt umber pileus, wider basidia (20 × 5–7 μm in *H. cumulatum* vs. 14–21 × 3–4 μm), and larger basidiospores [4–5.5 × 4–5 μm in *H. cumulatum* vs. (3.5–)4–5 × 3–4.5(–5) μm in *H. radiatum*, Harrison, 1964].

*Hydnellum* species tend to grow in moist woodlands with thick mosses, under pine needles or oak leaves, which help to reduce water loss. Most of the specimens were collected from Pinaceae, Fagaceae forests, or mixed forests (Table 2). But the Fagaceae forests were regarded as the preference for *Hydnellum* by comparing the frequency of the collection sites (Table 2). It indicated that species in *Hydnellum* are host-biased, which can be used as an auxiliary basis for species discovery and identification.

The combination of the traditionally morphological observation and molecular systematics methods can objectively reveal the diversity and phylogenetic relationship of *Hydnellum* species. Few studies were using phylogenetic analyses of *Hydnellum* in the past, and most of them were only based on the ITS sequences of several species (Ainsworth et al., 2010; Baird et al., 2013; Loizides et al., 2016). Mu et al. (2021) conducted a phylogenetic analysis of *Hydnellum* and *Sarcodon* based on 4-gene sequences (ITS + nLSU + nSSU + RPB2), which undoubtedly filled in the blank of multiple gene fragments of *Hydnellum*. In this study, the phylogeny of *Hydnellum* was carried out based on four gene markers. In this study, both ITS + LSU and ITS + LSU + SSU + RPB2 datasets share a similar topology with Mu et al. (2021), but with discrepant bootstrap values.

Combining with the macro- and micro-morphological observation and scanning electron microscope shot, the number of *Hydnellum* species has been expanded to 22 in China, and it indicated that more potential species of *Hydnellum* could be discovered by combined evidence of morphological characters, molecular data, and ecological habits. Moreover, the sequences of the largest subunit of the RNA polymerase II (RPB1) gene of *Hydnellum* were also provided in this study, which might be useful for future phylogenetic studies. The primer pairs RPB1-Af and RPB1-Cr for the RPB1 gene used in this study are the same as in previous studies (White et al., 1990; Liu et al., 2022b). However, there are still many species of *Hydnellum* lacking molecular data, which limits the systematic study of this genus. For the time being, the common gene marker for the identification of most *Hydnellum* species is ITS, while more terminal nodes in phylogenetic trees are needed to investigate by using more gene markers, such as RPB1 and RPB2. There are only 22 RPB2 sequences and no RPB1 sequence of *Hydnellum* in NCBI (Accessed 7 May 2022)^2^. It is necessary to obtain diverse molecular sequences to build a more scientific system between the species and genera in hydnoid stipitate fungi.

## Data availability statement

The datasets presented in this study can be found in online repositories. The names of the repository/repositories and accession number(s) can be found below: https://www.ncbi.nlm.nih.gov/genbank/, ON603638-ON603665 and https://www.ncbi.nlm.nih.gov/genbank/, ON605658-ON605669.

## Author contributions

B-KC designed the research. B-KC, Y-FS, SL, T-MX, D-MW, NG, and C-GS prepared the samples. C-GS, SL, and T-MX conducted the molecular experiments and analyzed the data. C-GS, Y-FS, D-MW, NG, and B-KC drafted the manuscript. All authors read and agreed to the published version of the manuscript.

## Footnotes

1
http://www.indexfungorum.org/

2
https://www.ncbi.nlm.nih.gov/protein
